# Robust Baseline-Free Damage Localization by Using Locally Perturbed Dynamic Equilibrium and Data Fusion Technique

**DOI:** 10.3390/s20205964

**Published:** 2020-10-21

**Authors:** Shancheng Cao, Huajiang Ouyang, Chao Xu

**Affiliations:** 1 School of Astronautics, Northwestern Polytechnical University, Xi’an 710072, China; chao_xu@nwpu.edu.cn; 2 Department of Mechanical, Materials and Aerospace Engineering, School of Engineering, The University of Liverpool, Liverpool L69 3GH, UK; h.ouyang@liverpool.ac.uk

**Keywords:** damage localization, singular value decomposition, power spectral density transmissibility, operational modal analysis, pseudo-excitation method

## Abstract

Mode shape-based structural damage identification methods have been widely investigated due to their good performances in damage localization. Nevertheless, the evaluation of mode shapes is severely affected by the measurement noise. Moreover, the conventional mode shape-based damage localization methods are normally proposed based on a certain mode and not effective for multi-damage localization. To tackle these problems, a novel damage localization approach is proposed based on locally perturbed dynamic equilibrium and data fusion approach. The main contributions cover three aspects. Firstly, a joint singular value decomposition technique is proposed to simultaneously decompose several power spectral density transmissibility matrices for robust mode shape estimation, which statistically deals better with the measurement noise than the traditional transmissibility-based methods. Secondly, with the identified mode shapes, an improved pseudo-excitation method is proposed to construct a baseline-free damage localization index by quantifying the locally damage perturbed dynamic equilibrium without the knowledge of material/structural properties. Thirdly, to circumvent the conflicting damage information in different modes and integrate it for robust damage localization, a data fusion scheme is developed, which performs better than the Bayesian fusion approach. Both numerical and experimental studies of cantilever beams with two cracks were conducted to validate the feasibility and effectiveness of the proposed damage localization method. It was found that the proposed method outperforms the traditional transmissibility-based methods in terms of localization accuracy and robustness.

## 1. Introduction

Structural damage identification, aiming at detecting and assessing the structural damage at or near its onset during operation, plays a significant role in maintaining the safety and reliability of civil and mechanical structures [1,2,3]. One of the major challenges in this field is that damage is a local phenomenon that naturally appears and propagates in a small region, which presents a great difficulty in detection [4,5,6]. Therefore, an effective damage identification approach should be local in nature [7,8,9]. Differently from the traditional vibration-based damage identification methods that use global damage features, a novel damage localization approach is proposed in this paper based on the estimated mode shapes to examine the damage-induced local dynamic disturbances. However, two major issues, that is, noise-robust mode shape estimation and effective damage localization index, hamper its practical engineering applications, which will be discussed and tackled in this investigation.

When compared with natural frequencies, mode shape-based damage identification methods are more sensitive to local damage while being less sensitive to environmental variability [10,11]. Nevertheless, the evaluation of mode shapes is vulnerable to measurement uncertainties, as the data acquisition at discrete spatial points can be readily contaminated by the measurement noise [12,13]. As for mode shape-based damage identification, it is desirable to obtain mode shapes via operational modal analysis (OMA), as excitation forces are normally unavailable or impossible to be acquired under operational conditions [14,15,16]. A comprehensive review of different OMA methods was summarized and presented by Rainieri and Fabbrocino [17]. Stochastic subspace identification (SSI) and frequency domain decomposition (FDD) are two popular OMA approaches that are widely used [18]. Recently, the second-order blind source identification (SOBI) has attracted much more attention, which evaluates the modal parameters based on the concepts of sources and mixing matrix [19]. In addition, Yuen and Au [20] proposed a Bayesian operational modal analysis, which could output the uncertainty quantification of estimated modal parameters. Nevertheless, a major shortcoming of those OMA methods is that the random (white noise) excitation assumption is adopted, which is not physically true in practical engineering. To overcome this limitation, identification of modal parameters based on transmissibility measurements has been widely investigated, which works without any assumption regarding to the nature of excitation forces [21,22]. Araújo and Laier [23] adopted an excitation force in the form of colored noise with a predominant frequency of 12 Hz to demonstrate the effectiveness of the transmissibility-based OMA.

For operational modal analysis, mode shapes are typically estimated by decomposing a matrix or some linear combinations of matrices, such as power spectral density (PSD) and covariance matrices. However, those matrices do not exactly possess identical eigen-structure because of limited measurement data and various uncertainties. To circumvent this, a kind of common eigen-structure is proposed in this paper. In this method, several power spectral density transmissibility (PSDT) matrices are simultaneously diagonalized via a joint singular value decomposition (SVD) approach for robust mode shape estimation, which is the first contribution of this paper.

Apart from the robust mode shape estimation problem, another critical issue is how to quantify the damage-induced local dynamic disturbances based on the mode shapes of damaged states for damage localization. Originally, the evaluation of a locally perturbed dynamic equilibrium, also known as the pseudo-excitation (PE) method, was developed to tackle the local force identification problem [24,25]. As for damage identification, the damage index construction for an impaired structural component can be considered as equivalent to the computation of PE forces on its pristine counterpart [26,27,28]. However, the damage localization index constructed in PE method is based on the local dynamic equilibrium, thereby inheriting several shortcomings. Firstly, some material or structural properties in the equation of local motion, such as stiffness and cross-sectional area, may be inaccurately described or even unknown a priori [29]. Secondly, a local dynamic equilibrium with the assumption of no damping effects is typically adopted, which is limited to non-resonant frequencies [30].

Motivated by addressing the aforementioned issues of the PE method, the current work proposes a comprehensive method, which is the second contribution of this paper. In this method, a local dynamic equilibrium model considering viscous damping is defined and statistically evaluated to establish the damage localization index without requiring the knowledge of material/structural parameters, thereby extending the PE method to be applied under both resonant and non-resonant conditions. Consequently, the identified mode shapes by the transmissibility-based OMA can be used in the PE method for damage identification. Here, the estimated mode shape data can be treated as equivalent to the normalized displacement data. Moreover, it is impossible to localize all the damage positions by using a single mode shape, as the sensitivity of mode shapes to damage depends on the damage locations. Therefore, a data fusion approach inspired by Bayesian fusion is proposed and investigated to effectively combine the damage information of different modes for robust damage localization, which is the third contribution of this paper.

The structure of this paper is as follows. In Section 2, a joint SVD approach is proposed to simultaneously decompose several PSDT matrices for robust mode shape estimation. Moreover, an enhanced PE method is developed in Section 3 to construct an effective damage localization index, which considers the damping effects and does not require the knowledge of the material/structural properties. In addition, to integrate the PE-based damage location information in different modes, a data fusion strategy that was inspired by Bayesian fusion is proposed in Section 4, which overcomes the conflicting damage location information. Numerical and experimental studies are presented to verify the proposed damage localization method in Section 5 and Section 6, respectively. Finally, some key conclusions are summarized in Section 7.

## 2. Robust Mode Shape Estimation via Transmissibility-Based OMA

### 2.1. Traditional Transmissibility-Based OMA

In operational modal analysis, the estimation of mode shapes or operational deflection shapes requires a set of spatial measurement points. Here, the measurement vector $y\left(t\right)\in R^{m\times 1}$ is assumed to be acquired at m measurement points. Without the information of inputs, a general assumption that the system is subjected to n external excitations is adopted.

The PSDT $T_{ij}^{k}\left(\omega \right)$ between outputs$\;y_{i}\left(t\right)$ and$\;y_{j}\left(t\right)$ with reference to another output $y_{k}\left(t\right)$ is defined as the ratio of cross PSD $S_{ik}\left(\omega \right)$ and $S_{jk}\left(\omega \right)$, which is written as

(1) $$T_{ij}^{k}\left(\omega \right)=S_{ik}\left(\omega \right)/S_{jk}\left(\omega \right)$$

where i,j,k indicate different locations of output responses on a structure; $S_{ik}\left(\omega \right)$ represents the cross PSD between output $y_{i}\left(t\right)$ and $y_{k}\left(t\right)$.

Equation (1) implies that the PSDT function does not require the information of excitation forces. Therefore, the PSDT method is suitable for output-only analysis and input-output modal analysis. For stationary stochastic vibration, the relationship between the input and the output PSD matrices is in the form of

(2) $$S_{yy}\left(\omega \right)=H\left(\omega \right)S_{ff}\left(\omega \right)H{\left(\omega \right)}^{*}$$

where $S_{yy}\left(\omega \right)\in R^{m\times m}$ and $S_{ff}\left(\omega \right)\in R^{n\times n}$ are the PSD matrices of the responses and inputs (excitation forces), respectively; $H\left(\omega \right)\in R^{m\times n}$ indicates the FRF matrix; and $H{\left(\omega \right)}^{*}$ represents the Hermitian transpose of $H\left(\omega \right)$. Therefore, the cross PSD $S_{ik}\left(\omega \right)$ can be expressed as $S_{ik}\left(\omega \right)=\sum_{p=1}^{n}\sum_{q=1}^{n}H_{iq}\left(\omega \right)S_{qp}\left(\omega \right)H_{kp}{\left(\omega \right)}^{*}$ and the PSDT in Equation (1) can be rewritten as

(3) $$T_{ij}^{k}\left(\omega \right)=\frac{\sum_{p=1}^{n}\sum_{q=1}^{n}H_{iq}\left(\omega \right)S_{qp}\left(\omega \right)H_{kp}{\left(\omega \right)}^{*}}{\sum_{p=1}^{n}\sum_{q=1}^{n}H_{jq}\left(\omega \right)S_{qp}\left(\omega \right)H_{kp}{\left(\omega \right)}^{*}}$$

where $H_{iq}\left(\omega \right)=\sum_{r=1}^{n_{m}}\Phi_{ir}\Phi_{qr}/\left(\omega_{r}^{2}-\omega^{2}+i2\xi \omega \omega_{r}\right)$ is the FRF between the output $y_{i}\left(t\right)$ and the input $f_{q}\left(t\right)$ with $n_{m}$, ξ and $\Phi_{ir}$ denoting the number of modes, damping ratio and the mode shape value at location i for the r-th mode, respectively; $S_{qp}\left(\omega \right)$ represents the cross PSD between input $f_{q}\left(t\right)$ and input $f_{p}\left(t\right)$ with p and q indicating the locations of the input excitation forces. 

When the frequency of excitation ω approaches the r-th natural frequency, the dynamic response is dominated by the contribution of this r-th mode and the contributions of other vibration modes can be negligible for a structure with well separated modes and small damping ratios [23]. Therefore, $H_{iq}\left(\omega \right)$ can be well approximated by $\Phi_{ir}\Phi_{qr}/\left(\omega_{r}^{2}-\omega^{2}+i2\xi \omega \omega_{r}\right)$ near the r-th natural frequency. Consequently, the expression of cross PSD $S_{ik}\left(\omega \right)$ and $S_{jk}\left(\omega \right)$ at a natural frequency $\omega_{r}$ can be approximated as

(4) $$\begin{array}{c} \underset{\omega \to \omega_{r}}{\lim}S_{ik}\left(\omega \right)≅\Phi_{ir}\sum_{p=1}^{n}\sum_{q=1}^{n}{\hat{H}}_{iq}\left(\omega \right)S_{qp}\left(\omega \right)H_{kp}{\left(\omega \right)}^{*}\\ \underset{\omega \to \omega_{r}}{\lim}S_{jk}\left(\omega \right)≅\Phi_{jr}\sum_{p=1}^{n}\sum_{q=1}^{n}{\hat{H}}_{jq}\left(\omega \right)S_{qp}\left(\omega \right)H_{kp}{\left(\omega \right)}^{*} \end{array}$$

where ${\hat{H}}_{iq}\left(\omega \right)=\Phi_{qr}/\left(\omega_{r}^{2}-\omega^{2}+i2\xi \omega \omega_{r}\right)$, which does not involve the output information at location i. Similarly, for cross PSD $S_{jk}\left(\omega \right)$, ${\hat{H}}_{jq}\left(\omega \right)=\Phi_{qr}/\left(\omega_{r}^{2}-\omega^{2}+i2\xi \omega \omega_{r}\right)$. Thus, ${\hat{H}}_{iq}\left(\omega \right)={\hat{H}}_{jq}\left(\omega \right)$. In this case, $T_{ij}^{k}\left(\omega \right)$ in Equation (3) will converge to $\Phi_{ir}/\Phi_{jr}$ when ω approaches the r-th natural frequency

(5) $$\underset{\omega \to \omega_{r}}{\lim}T_{ij}^{k}\left(\omega \right)=\underset{\omega \to \omega_{r}}{lim}\frac{\Phi_{ir}\sum_{p=1}^{n}\sum_{q=1}^{n}{\hat{H}}_{iq}\left(\omega \right)S_{qp}\left(\omega \right)H_{kp}{\left(\omega \right)}^{*}}{\Phi_{jr}\sum_{p=1}^{n}\sum_{q=1}^{n}{\hat{H}}_{jq}\left(\omega \right)S_{qp}\left(\omega \right)H_{kp}{\left(\omega \right)}^{*}}=\frac{\Phi_{ir}}{\Phi_{jr}}$$

Moreover, the PSDT matrix $T_{j}\left(\omega \right)$ is assembled by PSDT $T_{ij}^{k}\left(\omega \right)$ with different output point i and reference output point k as

(6) $$T_{j}\left(\omega \right)=\left[\begin{array}{ccc} T_{1j}^{1} & \begin{array}{ccc} \cdots & T_{1j}^{k} & \cdots \end{array} & T_{1j}^{m}\\ \begin{array}{c} \vdots \\ T_{ij}^{1}\\ \vdots \end{array} & \begin{array}{c} \begin{array}{ccc} \cdots & \vdots & \cdots \end{array}\\ \begin{array}{ccc} \cdots & T_{ij}^{k} & \cdots \end{array}\\ \begin{array}{ccc} \cdots & \vdots & \cdots \end{array} \end{array} & \begin{array}{c} \vdots \\ T_{ij}^{m}\\ \vdots \end{array}\\ T_{mj}^{1} & \begin{array}{ccc} \cdots & T_{mj}^{k} & \cdots \end{array} & T_{mj}^{m} \end{array}\right],j=1,2,\cdots ,m$$

It is worth noting that the transmissibility $T_{ij}^{k}\left(\omega \right)$ for different reference points such as $T_{ij}^{k_{1}}\left(\omega \right)$ and $T_{ij}^{k_{2}}\left(\omega \right)$ will converge to the same ratio of amplitudes of mode shapes at corresponding natural frequencies. Thus, when approaching the r-th natural frequency, the columns of the PSDT $T_{j}\left(\omega \right)$ will be identical with each other, given by

(7) $$\underset{\omega \to \omega_{r}}{\lim}T_{j}\left(\omega \right)=\frac{1}{\Phi_{jr}}\left[\begin{array}{ccc} \begin{array}{cc} \Phi_{1r} & \Phi_{1r}\\ \Phi_{2r} & \Phi_{2r} \end{array} & \cdots & \begin{array}{c} \Phi_{1r}\\ \Phi_{2r} \end{array}\\ \begin{array}{cc} \vdots & \vdots \end{array} & \ddots & \vdots \\ \begin{array}{cc} \Phi_{mr} & \Phi_{mr} \end{array} & \cdots & \Phi_{mr} \end{array}\right],j=1,2,\cdots ,m$$

From Equation (7), it indicates that the column rank of $T_{j}\left(\omega_{r}\right)$ will be one at a certain natural frequency. With this property, the system natural frequencies can by identified through a traditional approach [31], which is

(8) $$\Delta T^{-1}\left(\omega \right)=\sum_{j=1,j\neq i}^{m}\sum_{i=1}^{m}\left(\sum_{k_{1}=1,k_{1}\neq k_{2}}^{m}\sum_{k_{2}=1}^{m}\frac{1}{\left|T_{ij}^{k_{1}}\left(\omega \right)-T_{ij}^{k_{2}}\left(\omega \right)\right|}\right)$$

At an identified natural frequency, Equation (7) is normally processed by SVD method to evaluate the singular vector corresponding to the largest singular value as a good estimate of the mode shape [23]. 

### 2.2. The Proposed Transmissibility-Based OMA by Using Joint SVD

However, the identification approach based on Equation (8) tends to introduce some additional false natural frequencies [32]. To overcome this, a novel approach is proposed to evaluate the natural frequencies based on the SVD of PSDT matrices. $T_{j}\left(\omega \right)$ is decomposed by singular value decomposition as

(9) $$T_{j}\left(\omega \right)=U_{j}\left(\omega \right)D_{j}\left(\omega \right)V_{j}{\left(\omega \right)}^{*}$$

where $D_{j}\left(\omega \right)$ is a diagonal matrix with non-negative singular values in a descending order $\sigma_{j1}\geq \sigma_{j2}\geq \cdots \geq \sigma_{jm}$. At a certain natural frequency, the rank of $T_{j}\left(\omega \right)$ should be one in theory and practically the first singular value will be much larger than all the other singular values. This can be reflected by using the ratio$\;\delta_{1,l}^{j}\left(\omega \right)=\sigma_{j1}/\sigma_{jl}$ (l=2,3,⋯,m), and a system natural frequency indicator $\gamma \left(\omega \right)$ is proposed by a combination of singular values as

(10) $$\gamma \left(\omega \right)=\overset{m}{\underset{l=2}{\prod }}\delta_{1,l}^{j}$$

where Π indicates the multiplication operator. To reduce the effects of measurement noise, the last several smallest singular values are not suggested to be used in Equation (10). 

Moreover, in a narrow frequency band ($\omega_{r1}\leq \omega_{r}\leq \omega_{r2}$) around a natural frequency$\;\omega_{r}$, the rank of PSDT matrix $T_{j}\left(\omega \right)$ is still almost 1 and the mode shape $\varphi_{r}$ is the dominant mode as well. Thus, the dominant mode shape $\varphi_{r}$ can be estimated by applying joint SVD to the PSDT matrices of this frequency band ($\omega_{r1}\leq \omega_{r}\leq \omega_{r2}$):

(11) $$T_{j}\left(\omega_{r+kk}\right)=U_{r}D_{j}\left(\omega_{r+kk}\right)V_{r}{}^{*}+E_{j}\left(\omega_{r+kk}\right),kk=-K,-K+1,\cdots ,K$$

where the joint unitary diagonalizers $U_{r}\;$ and $V_{r}$ are identical but diagonal matrix $D_{j}\left(\omega_{r+kk}\right)$ and noise matrix$\;E_{j}\left(\omega_{r+kk}\right)$ are different at each kk. A traditional approach of solving the joint SVD is the least-squares method, in which the over-determined decomposition is treated as a minimization problem of variables$\;U_{r}$**,** $V_{r}$ and $D_{j}\left(\omega_{r+kk}\right)$:

(12) $$J\left(U_{r},V_{r},D_{j}\right)=\overset{K}{\underset{kk=-K}{\sum }}\|T_{j}\left(\omega_{r+kk}\right)-U_{r}D_{j}\left(\omega_{r+kk}\right)V_{r}{}^{*}\|$$

There are several numerically efficient algorithms for solving Equation (12), such as power iterations, Givens rotations and matrix gradient flows [33]. In this study, the joint SVD problem is readily addressed via a joint approximate diagonalization (JAD) approach based on Givens rotations. In the regime of JAD, the estimation of $U_{r}$ in Equation (12) is transformed to minimize the following function as

(13) $$J_{1}\left(U_{r},D_{j}^{1}\right)=\overset{K}{\underset{kk=-K}{\sum }}\|T_{j}\left(\omega_{r+kk}\right)T_{j}{\left(\omega_{r+kk}\right)}^{*}-U_{r}D_{j}^{1}\left(\omega_{r+kk}\right)U_{r}{}^{*}\|$$

By solving Equation (13), the mode shape at natural frequency $\omega_{r}$ is the column of $U_{r}$ which corresponds to the largest diagonal element in $D_{j}^{1}\left(\omega_{r}\right)=D_{j}\left(\omega_{r}\right)D_{j}^{*}\left(\omega_{r}\right)$. It can be seen that joint SVD is an extension of the singular value decomposition to a set of more than two matrices. Furthermore, joint SVD is a more general tool for non-symmetric, possibly rectangular matrices than JAD method which is limited to Hermitian or symmetric matrix set. 

## 3. Damage Localization Based on the Improved PE Method

In this section, an improved PE method is proposed, which identifies the damage locations without the information of the unknown material/structural properties. Without loss of generality, the transverse vibration of a beam component is taken as an example and the equation of motion is given according to the Euler–Bernoulli beam theory as

(14) $$EI\frac{\partial^{4}w\left(x,t\right)}{\partial x^{4}}+C\frac{\partial w\left(x,t\right)}{\partial t}+\rho A\frac{d^{2}w\left(x,t\right)}{\partial t^{2}}=f\left(x,t\right)$$

where $w\left(x,t\right)$ and $f\left(x,t\right)$ are the transverse displacement and transverse distributed load at location x, respectively; E, I, C, ρ and A represent the Young’s modulus, the second moment of the cross-sectional area, damping coefficient, mass density and cross-sectional area, respectively.

Specifically, in a harmonic regime, the steady-state vibration $w\left(x,t\right)$ can be written as $W\left(x\right)e^{i\omega t}$. Assuming that the beam has a uniform cross-section and constant material properties within the inspection area, Equation (14) can be expressed under harmonic regime as

(15) $$EI\frac{\partial^{4}W\left(x\right)}{\partial x^{4}}+iC\omega W\left(x\right)-\rho A\omega^{2}W\left(x\right)=f\left(x,\omega \right)$$

In Equation (15), without the external excitation, the right-hand side becomes zero for a pristine beam. Nevertheless, with the occurrence of damage in this beam component, the left-hand side of Equation (15) does not equal zero anymore when $f\left(x,\omega \right)=0$, which can be adopted as a damage index (DI).

(16) $$\begin{array}{c} DI\left(x,\omega \right)=EI\nabla^{4}W\left(x\right)+iC\omega W\left(x\right)-\rho A\omega^{2}W\left(x\right)\\ \left[\left(EI-\Delta_{EI}\right)\nabla^{4}W\left(x\right)+i\left(C-\Delta_{c}\right)\omega W\left(x\right)-\left(\rho A-\Delta_{\rho A}\right)\omega^{2}W\left(x\right)\right]\\ +\left[\Delta_{EI}\nabla^{4}W\left(x\right)+i\Delta_{c}\omega W\left(x\right)-\Delta_{\rho A}\omega^{2}W\left(x\right)\right] \end{array}$$

where $\Delta_{EI}$, $\Delta_{c}$ and $\Delta_{\rho A}$ represent the damage-caused changes in the structural properties corresponding to its stiffness, damping and mass, respectively; $\nabla^{4}W\left(x\right)$ becomes $\frac{\partial^{4}W\left(x\right)}{\partial x^{4}}$ for a beam with $\nabla^{4}$ denoting the double Laplacian operator. Due to the local dynamic equilibrium of the damaged beam component, Equation (16) can be rearranged as

(17) $$\begin{array}{c} \left(EI-\Delta_{EI}\right)\nabla^{4}W\left(x\right)+i\left(C-\Delta_{c}\right)\omega W\left(x\right)-\left(\rho A-\Delta_{\rho A}\right)\omega^{2}W\left(x\right)=0\\ DI\left(x,\omega \right)=\Delta_{EI}\nabla^{4}W\left(x\right)+i\Delta_{c}\omega W\left(x\right)-\Delta_{\rho A}\omega^{2}W\left(x\right) \end{array}$$

From Equations (16) and (17), it can be seen that the transverse vibration of a damaged beam component is equivalent to its corresponding pristine counterpart subjected to a pseudo-excitation force [25,30]. Consequently, the damage-induced local pseudo-excitation force can be harnessed for damage detection, localization and quantification.

However, the values of EI, C or ρA in Equation (16) are normally unavailable or inaccurately described in practice. Instead of evaluating the individual material or geometric parameters, an integrated parameter is proposed by converting Equation (16) into

(18) $$DI\left(x,\omega \right)=\nabla^{4}W\left(x\right)+c_{0}W\left(x\right)$$

where $c_{0}=\left(iC\omega -\rho A\omega^{2}\right)/EI$ is a constant value at a given ω. Provided that damage zones only occupy a small area of the inspected structure, the majority of the structure still satisfies $DI\left(x,\omega \right)=0$ within the inspection region when $f\left(x,\omega \right)=0$. Therefore, coefficient $c_{0}$ can be readily determined based on the least-squares criterion at each interested ω. In this case, the proposed DI in Equation (18) is capable of localizing damage without the knowledge of material/structural properties. 

## 4. Robust Damage Localizations Based on a Novel Data Fusion Approach

Naturally, for any given ω, the sensitivity of $DI\left(x,\omega \right)$ to damage depends on damage locations. Hence, an integrated damage index that incorporates damage-induced characteristics at different modes should be more robust and effective. However, the damage location information contained in different modes is often in conflict with each other. For instance, one mode provides damage location evidence at position i, while another mode may suggest somewhere else. Therefore, a novel data fusion approach is proposed in this paper to circumvent the conflicting damage location information for robust damage localization. Before introducing the proposed data fusion method, a review of the Bayesian fusion is presented first.

At a given ω, the damage probability of each measured point is defined as

(19) $$P\left(x_{i},\omega \right)=P\left(\omega |x_{i}\right)=\frac{DI^{2}\left(x_{i},\omega \right)}{\sum_{k=1}^{m}DI^{2}\left(x_{k},\omega \right)}$$

The basic probabilities at different angular frequencies can be combined based on the Bayesian fusion. Consider that there are two damage information sources at $\omega_{1}$ and $\omega_{2}$. According to the Bayesian formula, the combination of two damage information sources is

(20) $$P\left(x_{i}|\omega_{1},\omega_{2}\right)=\frac{P\left(\omega_{1},\omega_{2}|x_{i}\right)P\left(x_{i}\right)}{\sum_{k=1}^{m}P\left(\omega_{1},\omega_{2}|x_{k}\right)P\left(x_{k}\right)}$$

where the prior probability values are assumed as $P\left(x_{i}\right)=1/m$ $\left(i=1,2,\cdots ,m\right)\;$[34]. Furthermore, when each damage location information can be treated as independent, Equation (20) is expressed as

(21) $$P\left(x_{i}|\omega_{1},\omega_{2}\right)=\frac{P\left(\omega_{1}|x_{i}\right)P\left(\omega_{2}|x_{i}\right)P\left(x_{i}\right)}{\sum_{k=1}^{m}P\left(\omega_{1}|x_{k}\right)P\left(\omega_{2}|x_{k}\right)P\left(x_{k}\right)}$$

Similarly, the Bayesian fusion of damage information at M angular frequencies is

(22) $$P\left(x_{i}|\omega_{1},\omega_{2},\cdots ,\omega_{M}\right)=\frac{P\left(x_{i}\right)\prod_{r=1}^{M}P\left(\omega_{r}|x_{i}\right)}{\sum_{k=1}^{m}P\left(x_{k}\right)\prod_{r=1}^{M}P\left(\omega_{r}|x_{k}\right)}$$

From Equation (22), if damage information is not present at a source ω, the Bayesian fusion will not be able to provide effective damage localization, as individual damage information will be disappear in the multiplication operator. Thus, to circumvent this drawback of Bayesian fusion, a variant form of Bayesian fusion is proposed in this paper for robust damage localization, which is defined as

(23) $$P\left(x_{i}|\omega_{1},\omega_{2},\cdots ,\omega_{M}\right)=\frac{P\left(x_{i}\right)\sum_{r=1}^{M}P\left(\omega_{r}|x_{i}\right)}{\sum_{k=1}^{m}P\left(x_{k}\right)\sum_{r=1}^{M}P\left(\omega_{r}|x_{k}\right)}$$

## 5. Numerical Study

**Figure 1:**
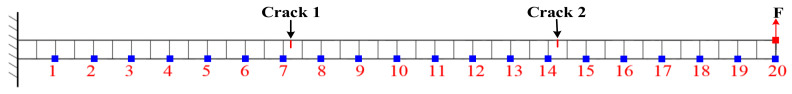
A cantilever beam with two open cracks.

A finite element model of a cantilever beam with two open cracks was coded based on the Euler–Bernoulli beam theory to validate the effectiveness of the proposed mode shape estimation method and the constructed damage localization index. Rayleigh damping, C=αM+βK (α=8.0272 and$\;\beta =1.0170\times 10^{-5}$, which sets a 2% damping ratio for the first and third modes), was utilized to include the damping effects. The beam was discretized into 40 elements, as shown in Figure 1, which is fine enough to provide a convergence solution. Other material and geometrical parameters of this cantilever beam are given in Table 1.

Moreover, the details about the two cracks are presented in Table 2 and the modelling of cracks is based on the fracture mechanics [35]. The stiffness matrix of intact elements is treated as unchanged while the stiffness matrix for a cracked element is defined as

(24) $$K_{c}=T_{s}{}^{T}G^{-1}T_{s},$$

in which the transformation matrix $T_{s}$ and flexibility matrix G of a cracked element are expressed as

(25) $$\begin{array}{c} T_{s}=\left[\begin{array}{c} -1,-l_{e},1,0\\ 0,-1,0,1 \end{array}\right]\\ G=\frac{1}{6EI}\left[\begin{array}{cc} 2l_{e}^{3} & 3l_{e}^{2}\\ 3l_{e}^{2} & 6l_{e} \end{array}\right]+\frac{18\pi \left(1-v^{2}\right)}{Ebh^{2}}\left[\begin{array}{cc} l_{e}^{2} & 2l_{e}\\ 2l_{e} & 4 \end{array}\right]\int_{0}^{h_{c}/h}\eta F_{Ι}^{2}\left(\eta \right)d\eta \end{array}$$

where $l_{e}$ denotes the element length, b is the beam width, h is the beam depth and $h_{c}$ is the depth of crack. $F_{I}\left(\eta \right)$ is an approximate expression of the mode-I stress intensity factor as

(26) $$F_{Ι}\left(\eta \right)=\sqrt{\frac{tan\left(\pi \eta /2\right)}{\pi \eta /2}}\frac{0.923+0.199{\left(1-sin\left(\pi \eta /2\right)\right)}^{4}}{cos\left(\pi \eta /2\right)},\eta =h_{c}/h$$

In addition, the random excitation force F possesses a normal distribution with the mean value and standard deviation being 0 and 50 N, respectively. Velocity time series are acquired at the labelled 20 points shown in Figure 1.

Firstly, while aiming to study the noise robustness of different mode shape estimation approaches, Gaussian white noise was introduced to contaminate the acquired velocity responses in the form of

(27) $${\hat{y}}_{i}\left(t\right)=y_{i}\left(t\right)+dn_{level}\sigma \left(y_{i}\left(t\right)\right)$$

where *d* implies a random value of normal distribution with a zero mean and variance being 1, $n_{level}$ is the noise level range of [0, 1] and $\sigma \left(y_{i}\left(t\right)\right)$ denotes the standard deviation of vibration responses at the i-th measurement point. To better represent the noise level $n_{level}$, it is quantified using signal-to-noise-ratio (SNR).

**Figure 2:**
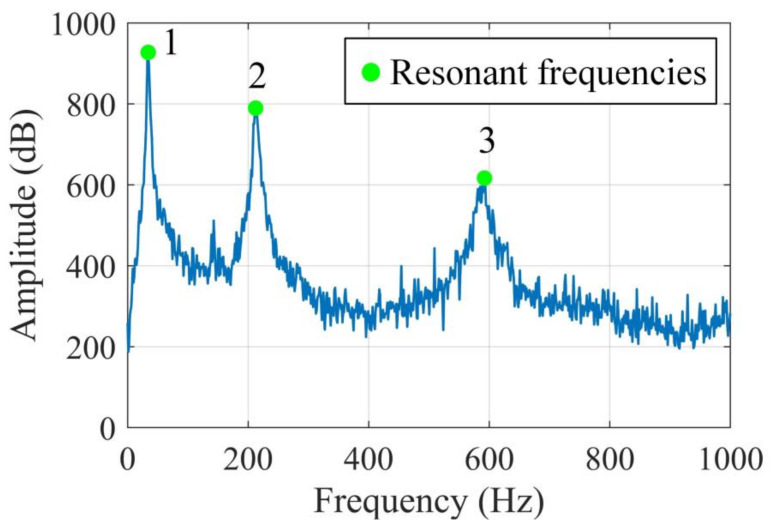
System natural frequency indicator based on the singular value decomposition (SVD) of the PSDT method.

**Figure 3:**
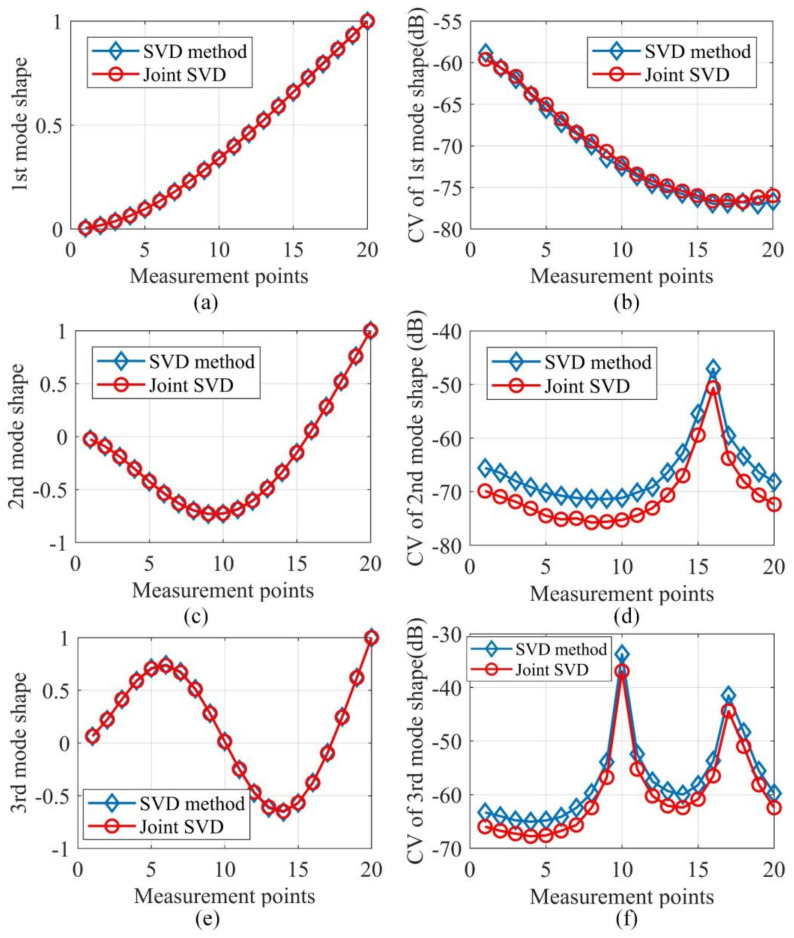
Estimated mode shapes and their CVs for the first three modes: (**a**) the 1st mode shape; (**b**) CV of the 1st mode shape; (**c**) the 2nd mode shape; (**d**) CV of the 2nd mode shape; (**e**) the 3rd mode shape; (**f**) CV of the 3rd mode shape.

The output responses were polluted by the same noise level SNR = 40 dB 1000 times. With each noise realization, the mode shapes were evaluated by SVD of PSDT and joint SVD of PSDT methods, respectively. For both SVD of PSDT and joint SVD of PSDT methods, the system natural frequencies were determined by the proposed natural frequency indicator in Equation (10), and examples of the identified natural frequencies of this numerical case are illustrated in Figure 2. The first three natural frequencies can be clearly detected, which demonstrates the effectiveness of the proposed system’s natural frequency indicator. With the obtained natural frequencies, their corresponding mode shapes were estimated by applying SVD of PSDT and joint SVD of PSDT methods, respectively. The first three mode shapes and their coefficients of variation (CVs) over 1000 noise realizations are shown in Figure 3. It is worth noting that the peaks in the CV plots in Figure 3d,f are located around the node points of corresponding mode shapes. The reason is that the mode shape values around node points are almost zero, which led to a very low signal-to-noise-ratio and large CV values.

**Figure 4:**
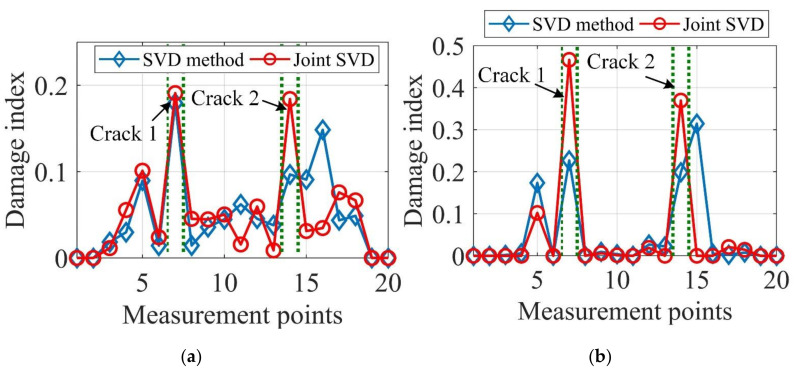
Damage localization results of two cracks with 5% depth reduction. (**a**) Proposed data fusion approach; (**b**) Bayesian fusion.

From Figure 3a,c,e, it can be seen that the estimated mode shapes by both methods are highly similar to each other. However, Figure 3d,f manifest that the CVs of the joint SVD method are smaller than those of the SVD method for the second and third modes, which shows that the mode shapes estimated by joint SVD of PSDT method are more noise-robust than those by SVD of PSDT method. Here, the CV of the first mode as given in Figure 3b indicates no obvious difference for these two mode shape estimation methods. The possible reason is that the first mode, which is the dominant mode shape of the random vibration in this study, is more robust to the influences of measurement noise than the mode shapes associated with higher natural frequencies. Furthermore, the proposed damage localization index based on identified mode shapes by joint SVD of PSDT should be more accurate and effective. For the validation of this conclusion, a numerical case containing two cracks of 5% depth reduction was studied and the damage localization results under noise level SNR = 40 dB are depicted in Figure 4.

In Figure 4, the damage localization results of the joint SVD of PSDT method outperform the SVD of PSDT method in terms of accuracy and noise robustness. In addition, by comparing Figure 4b with Figure 4a, it can be concluded that the proposed data fusion approach provides more accurate damage localization results than the traditional Bayesian fusion approach. Later on, the defects of the Bayesian fusion are further illustrated by using the experimental studies in Section 6.

**Figure 5:**
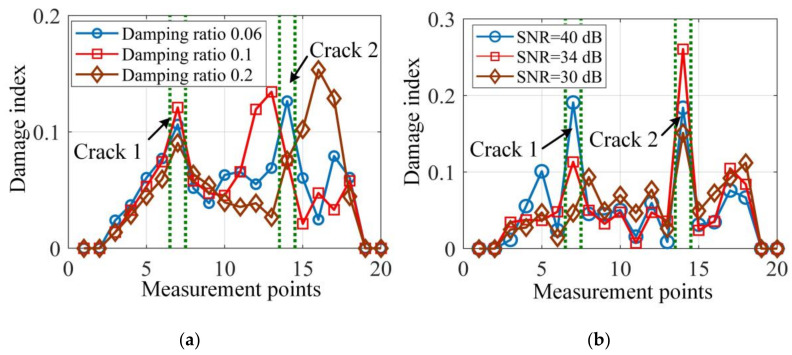
Damage localization results of two cracks with 5% depth reduction under (**a**) different damping ratios and (**b**) different noise levels.

**Figure 6:**
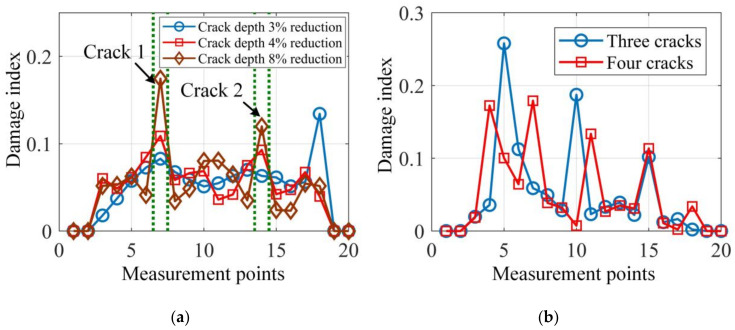
Nosie free damage localization results of (**a**) two cracks with different depth reductions and (**b**) different numbers of cracks with 10% depth reduction.

In addition, to test the sensitivity and robustness of the proposed damage localization method to different damping ratios, noise levels, severity of damage and damage positions, different damage scenarios were simulated and damage localization results are illustrated in Figure 5 and Figure 6.

In Figure 5a, the damage localization accuracy decreases for large damping ratios, but the damage index peaks still appear around the damage positions and provide useful damage localization information. The reason is that a higher damping ratio will degrade the estimation accuracy of resonant frequencies, and thus the corresponding mode shapes, as the resonant frequency peaks shown in Figure 2 will become flatter and harder to be identified in the proposed OMA method. For high noise levels, such as given in Figure 5b, the lower SNR undermines the damage localization results, as the two damage locations cannot correctly detected when SNR decreases to 30 dB. In addition, the two damage index peaks in Figure 5b have different heights, which indicates different damage sensitivities.

It can be seen from Figure 6a that the proposed damage localization method fails when the depth reduction of the two cracks gets as low as 3%. However, being able to identify depth reduction just above 3% should be considered to be highly accurate. Naturally, the larger the damage depth reduction, the sharper the damage index at the damage locations. In addition, the proposed method examines the local dynamic equilibrium point-by-point by using the mode shape data and it is suitable for multi-crack localization, as demonstrated in Figure 6b. In Figure 6b, the two damage cases with three and four cracks are clearly localized by the damage index peaks.

## 6. Experimental Studies

The purposes of this part were twofold. First, the mode shapes calculated by joint SVD of PSDT were experimentally proven to be more accurate and effective in damage localization than those by SVD of PSDT method. Secondly, the proposed data fusion approach was validated to be more robust and effective for damage localization than the Bayesian fusion.

**Figure 7:**
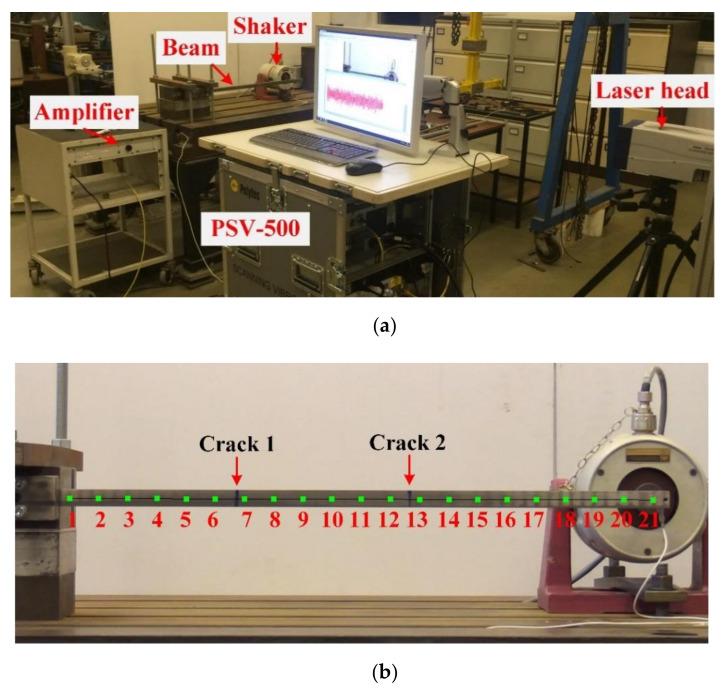
(**a**) Experimental set-up and (**b**) a cantilever beam with two cracks.

**Figure 8:**
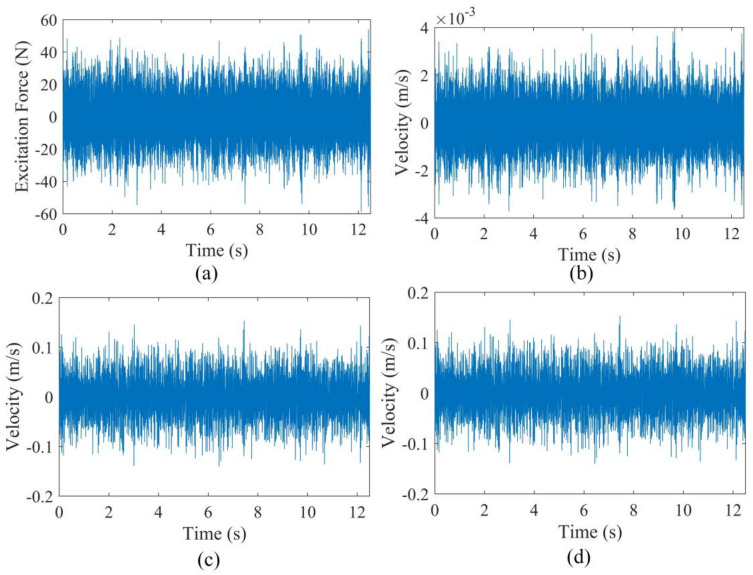
The acquired time domain signals of (**a**) excitation force, (**b**) measurement point 1, (**c**) measurement point 10 and (**d**) measurement point 21.

Two cantilever beams of $0.7\times 0.02\times 0.02\;m^{3}$ with two cracks of different damage severities were used. A PSV-500 Scanning Laser Vibrometer was used for the velocity response acquisition at the prescribed 21 measurement points shown in Figure 7b. A pseudo-random excitation with frequency range of 0–800 Hz was adopted to excite the cantilever beam at the free end via a shaker (LDS V406). The excitation has a normal distribution with mean value and standard deviation being 0 and 13.2 N, respectively. Furthermore, damage was machined as narrow slots, and its details are tabulated in Table 3. In addition, the cracks are on the opposite sides of the measurement surface and marked as the blue lines in the front view in Figure 7b. For each measurement point, as shown in Figure 7b, a total data acquisition time of 12.5 s was used with a sampling frequency of 2000 Hz. The PSV-500 system successively moved to the next measurement point while repeating the excitation. For information, the time domain signals of the excitation force and velocities at measurement points 1, 10 and 21 are shown in Figure 8.

**Figure 9:**
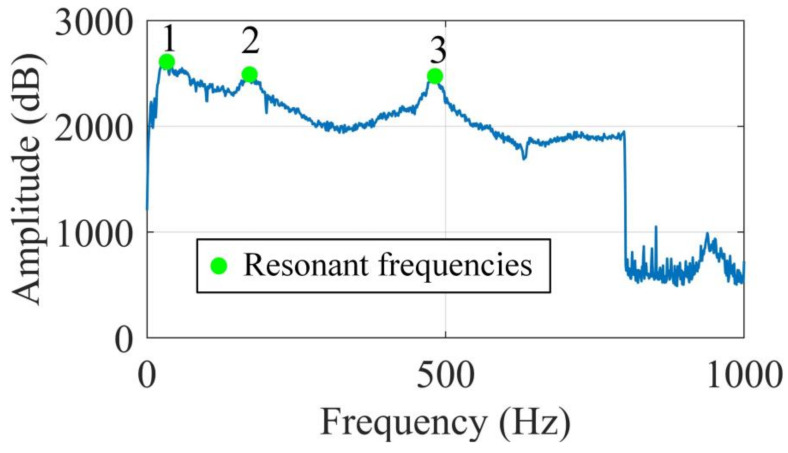
System natural frequency indicator based on the SVD of the PSDT method.

**Figure 10:**
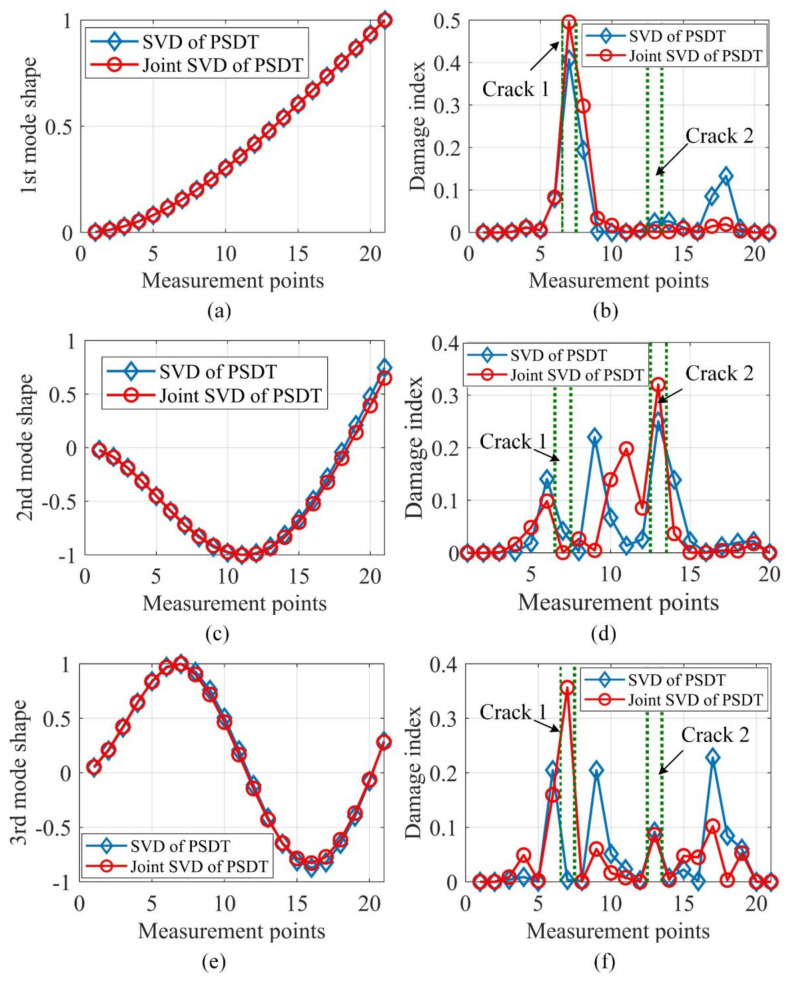
Estimated mode shapes and their individual damage localization results: (**a**) the 1st mode shape; (**b**) damage index of the 1st mode shape; (**c**) the 2nd mode shape; (**d**) damage index of the 2nd mode shape; (**e**) the 3rd mode shape; (**f**) damage index of the 3rd mode shape.

Firstly, the proposed system natural frequency indicator defined in Equation (10) is presented for experimental case 1 in Figure 9, and the first three natural frequencies can be clearly determined, which experimentally validates the effectiveness of the proposed system natural frequency indicator. After this, the corresponding mode shape and damage localization results at each natural frequency were computed by joint SVD of PSDT and SVD of PSDT methods respectively, which are illustrated in Figure 10.

**Figure 11:**
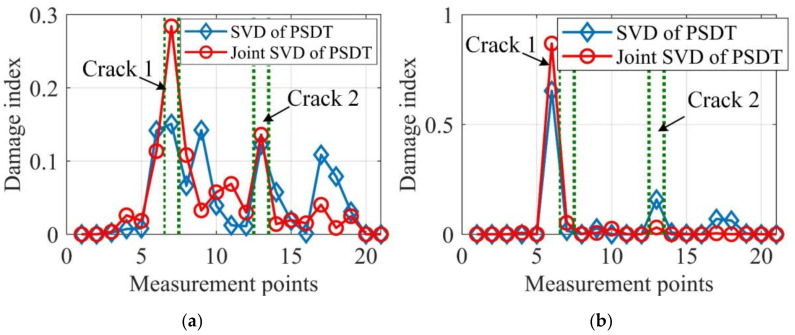
Integrated damage indexes of experimental case 1. (**a**) Proposed data fusion approach; (**b**) Bayesian fusion.

Figure 10b,d,f illustrates that the mode shape at a frequency is sensitive to damage, depending on locations. Therefore, a single mode shape is not robust for multi-damage localizations. Furthermore, Figure 10b,d,f validates that the mode shapes estimated based on joint SVD of PSDT matrices present more accurate damage localization results than those by SVD of PSDT matrix. To achieve a robust damage index, the Bayesian fusion and the proposed data fusion in Equation (23) are harnessed to construct the integrated damage localization index, whose results are graphed in Figure 11.

**Figure 12:**
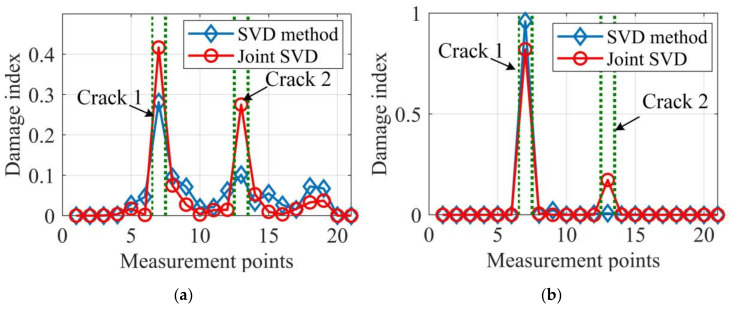
Integrated damage indexes of experimental case 2. (**a**) Proposed data fusion approach; (**b**) Bayesian fusion.

It can be seen from Figure 11 that damage localization results based on the joint SVD method are more accurate and provide fewer misleading alarms than those by the SVD method. Moreover, by comparing Figure 11b with Figure 11a, it can be concluded that damage localization based on the proposed data fusion approach outperforms the traditional Bayesian fusion method, as the damage localization of Bayesian fusion cannot correctly detect crack 1 and provides more misleading alarms. To further verify the proposed data fusion approach for robust damage localization, experimental case 2 with two cracks of 30% depth reduction was also tested and the damage localization results are presented in Figure 12.

A comparison of Figure 12a with Figure 12b shows that both the proposed data fusion approach and Bayesian fusion methods produce accurate damage localization results for the joint SVD method, but the Bayesian fusion cannot provide useful information for crack 2 for the SVD method. Besides, by considering the damage localization results in Figure 11, it can be concluded that the proposed data fusion approach always achieves accurate damage localization results and is more reliable, especially for less severe damage cases. Furthermore, the damage localization results based on the joint SVD method are always more accurate than those by SVD method. Therefore, the proposed mode shape estimation method and data fusion approach, when combined, significantly improve the damage localization accuracy that cannot be obtained based on the existing methods, and also have the potential to be applied in practical applications under ambient excitation.

## 7. Conclusions

This paper improves the accuracy of mode shape-based damage localization in three aspects: mode shape estimation, baseline-free damage index and data fusion. From both numerical and experimental perspectives, the proposed damage identification method was demonstrated to work effectively for beam-type structures without requiring the baseline-data in a pristine state. Moreover, the proposed method is naturally suitable for multi-crack localization, as it examines the local dynamic equilibrium point-by-point based on the estimated mode shapes. However, the proposed method performs poorly at high measurement noise levels and high damping ratios, as they degrade the estimation accuracy of mode shapes. Without the effects of measurement noise, the minimum damage that can be detected by the proposed method is about 4% depth reduction in the numerical simulation.

Other major conclusions are summarized as follows:

1. The joint SVD method was demonstrated to be more noise-robust in mode shape estimation than the traditional SVD method. The reason behind this is that mode shapes evaluating as the common eigen-structure of a set of matrices are more noise-robust than that just using a single matrix.
2. Mode shapes have their blind inspection zones, which are localized around their node points. Therefore, damage features of different mode shapes should be integrated to guarantee a robust and accurate damage localization.
3. The proposed transmissibility-based operational modal analysis method can provide robust estimation of natural frequencies and mode shapes without any assumption about the excitation force. Consequently, the proposed damage localization approach is promising for applications under various operational conditions.

## Figures and Tables

**Table 1:** Properties of cantilever beam.

Properties	Length (m)	Cross-Section $\left(m^{2}\right)$	Young’s Modulus (GPa)	Mass Density $\left(\mathrm{kg}/m^{3}\right)$	Poisson Ratio
Values	0.7	0.02 × 0.02	210	7850	0.33

**Table 2:** Crack information of the numerical study.

Cracks	Location (m)	Measurement Points	Depth Percentage
Crack 1	0.249	7~8	5%
Crack 2	0.499	14~15	5%

**Table 3:** Crack parameters of the two damage scenarios.

Cases	Cracks	Positions (m)	Measurement Points	Crack Depths (m)	Damage Percentage	Crack Widths (m)
1	Crack 1	0.2	6~7	0.004	20%	0.001
1	Crack 2	0.4	12~13	0.004	20%	0.001
2	Crack 1	0.2	6~7	0.006	30%	0.001
2	Crack 2	0.4	12~13	0.006	30%	0.001
